# miR-135a Alleviates Silica-Induced Pulmonary Fibrosis by Targeting NF-*κ*B/Inflammatory Signaling Pathway

**DOI:** 10.1155/2020/1231243

**Published:** 2020-06-17

**Authors:** Bin Xie, Can Lu, Chen Chen, Jianhua Zhou, Zhenghao Deng

**Affiliations:** ^1^Department of Pathology, Xiangya Hospital, Central South University, Changsha, Hunan 410008, China; ^2^Department of Pathology, School of Basic Medicine, Central South University, Changsha, Hunan 410013, China

## Abstract

Silica exposure triggers inflammatory response and pulmonary fibrosis that is a severe occupational or environmental lung disease with no effective therapies. The complicated biological and molecular mechanisms underlying silica-induced lung damages have not yet been fully understood. miR-135a inhibits inflammation, apoptosis, and cancer cell proliferation. But the roles of miRNA135a involved in the silica-induced lung damages remain largely unexplored. We investigated the roles and mechanisms of miR-135a underlying silica-induced pulmonary fibrosis. The present study showed silica exposure caused the decrease in miR-135a level but the increase in inflammatory mediators. Transduction of lentivirus expressing miR-135a reduced the level of inflammatory mediators in lung tissues from silica-treated mice and improved pulmonary fibrosis which was consistent with the downregulated *α*-SMA but enhanced E-cadherin. Moreover, miR-135a overexpression inhibited p-p65 level in lung tissues. Overexpression of miR-135a inhibitor strengthened TLR4 protein level and NF-*κ*B activation in BEAS-2B cells. Injection of PDTC, an inhibitor of NF-*κ*B, further reinforced miR-135a-mediated amelioration of inflammation and pulmonary fibrosis induced by silica. The collective data indicate miR-135a restrains NF-*κ*B activation probably through targeting TLR4 to alleviate silica-induced inflammatory response and pulmonary fibrosis.

## 1. Introduction

Exposure of silica (silicon dioxide) over several months can cause silicosis, a severe worldwide occupational or environmental lung disease with no effective therapies [1]. Epidemiological studies shows that the patients with silicosis are more likely to suffer tuberculosis and autoimmune disease [2, 3]. Inhalation of silica leads to the phagocytosis of free tiny particles by macrophages and the activation of epithelial cells in lung tissue which triggers the release of inflammatory mediators [4]. Inflammatory responses result in epithelial-mesenchymal transition (EMT) which stimulates fibroblast proliferation and the production of extracellular matrix (ECM) deposition, such as type I collagen (Col1) [5–8]. Excessive deposition of ECM causes progressive massive pulmonary fibrosis and impairment in respiratory functions [9]. The main histopathological characteristics underlying silicosis is quite investigated. However, the complicated biological and molecular mechanisms involved in lung damages have not yet been fully understood.

MicroRNAs (miRNAs), a class of endogenous short single-stranded noncoding RNAs of 22-25 nucleotides in length, are conserved in most eukaryotic cells. Primary miRNAs (pri-miRNAs) are initially transcribed from the genome and cleaved to precursors of miRNA (pre-miRNAs) containing about 70 nucleotides. These pre-miRNAs can be exported to the cytoplasm where they are further cleaved to generate mature miRNAs. miRNAs can assemble into RNA-induced silencing complex (RISC) and guide the enzyme-containing complex to target the messenger RNA (mRNA) sequence, leading to the downregulation of gene expression through the cleavage of their target mRNAs and/or inhibiting their translation. miRNAs derived from 1-5% of human genome are responsible for silencing more than 30% of protein-coding genes. Many noncoding miRNAs are involved in the physiological processes, and their abnormal expression affects several pathologies, including infectious diseases, cancer, and neurodegenerative diseases. Some evidence shows dysfunction of miRNAs in lung fibrosis [10, 11]. miR-135a exhibits downregulated expression in some diseases and cancers, and its upregulation can inhibit the inflammation, apoptosis, and cancer cell proliferation [12–14]. miR-135a also can suppress the cardiac fibrosis [15] but aggravates renal fibrosis in diabetic nephropathy [16]. miR-135a expression is increased in the lungs of antigen-exposed, sensitized mice [17]. miR-135a promotes inflammatory response in the lung tissues in monocrotaline- (MCT-) induced pulmonary arterial hypertension (PAH) in rats [18, 19] but inhibits migration and invasion in lung cancer cells [20]. However, the roles and molecular mechanisms of miR-135a in the silica-induced lung damages remain to be explored.

Phagocytosis of silica particles by macrophages is crucial for the inflammatory responses in the lung. The class B scavenger receptors (SR-B1) on the plasma membrane of macrophages recognize and tether silica particles then contribute to the subsequent canonical NLRP3 inflammasome activation [21] which requires not only nuclear factor *κ*B- (NF-*κ*B-) mediated upregulation of NLRP3 along with pro-IL-1b but also the assembly of multiple proteins including NLRP3, ASC, and pro-caspase-1 to activate caspase-1 [22, 23]. NF-*κ*B and inflammasome activation are required for the silica-induced pulmonary inflammation and lung fibrosis [24–26]. NF-*κ*B is a family of transcription factors and associates with inhibitors, I*κ*B proteins under unstimulated conditions. I*κ*B subunits confine NF-*κ*B to cytoplasm and inhibit it to bind the promoter region of target genes. As a rapid-acting transcription factor, the free NF-*κ*B subunit when separating from its inhibitors can translocate from the cytoplasm into the nucleus to promote gene transcription in response to stimuli or stress [27, 28]. The enhanced production of tumor necrosis factor-*α* (TNF-*α*), an inflammatory mediator which plays pivotal roles in the development of silica-induced lung damage [29], precedes the inflammasome activation in response to silica in lungs. Activation of TNF-*α* receptor is implicated in the ubiquitination and degradation of I*κ*B [30, 31]. Activated NF-*κ*B can bind to the promoter region of genes including TNF-*α* and collagens in lungs of mice exposed to silica [25, 32, 33]. Toll-like receptor 4 (TLR4) is a protein that in humans is encoded by the TLR4 gene. Activation of TLR4 results in activation of NF-*κ*B. Because NF-*κ*B plays important roles in the pathogenesis of silica-induced lung injury, it has been regarded as a potential target to weaken damage to lungs following silica exposure [26, 34, 35]. However, it is unclear whether miRNAs regulate the NF-*κ*B pathway in silica-induced pulmonary fibrosis.

In the present study, miR-135a was identified to be downregulated in lung tissues where the expression of inflammatory mediators were significantly increased following silica exposure. Transduction of the lentivirus expressing miR-135a decreased the expression of inflammatory mediators in lung tissues of silica-treated mice and improved the silica-induced pulmonary fibrosis. miR-135a overexpression in lung tissues reduced p-p65 and TLR4 following silica exposure. Moreover, silencing endogenous miR-135a significantly increased the TLR4 protein level and enhanced the activation of the NF-*κ*B pathway in BEAS-2B cells. In contrast, overexpression of miR-135a had opposite effects on TLR4 protein and activation of NF-*κ*B in BEAS-2B cells. Injection of PDTC, an inhibitor of NF-*κ*B, further enhanced the miR-135a-mediated improvement of inflammation and pulmonary fibrosis induced by silica. Taken together, our data indicate that miR-135a which is suppressed following silica inhalation alleviates the pulmonary fibrosis through targeting TLR4 to inhibit the NF-*κ*B pathway.

## 2. Materials and Methods

### 2.1. Animals

Male C57BL/6 mice were housed in the Laboratory Animal Facility of Hunan SJA Laboratory Animal Co., Ltd. All animal experiments were performed in accordance with the guideline for the Care and Use of Laboratory Animals. For induction of pulmonary fibrosis by silica exposure, C57BL/6 male mice (6-week old) were anesthetized using sodium pentobarbital (Sigma-Aldrich, USA) through intraperitoneal injection; then, the mice were intratracheally instilled with 50 *μ*l saline solution containing or no silica particles in suspension (50 mg/kg, Sigma-Aldrich, USA). The mice were sacrificed 4 weeks after silica instillation, and lung tissues were collected to be analyzed. For PDTC treatment, the mice were intraperitoneally injected with PDTC (100 mg/kg, Sigma-Aldrich, USA) or saline once daily from day 1 to day 21.

### 2.2. Hematoxylin and Eosin Staining and Assessment of Pulmonary Fibrosis

The lung tissues were fixed in 4% paraformaldehyde overnight at 4°C and then were embedded with paraffin wax. Five *μ*m thick sections were serially cut then stained using hematoxylin and eosin (H&E) to assess the severity of fibrosis. Briefly, the lung sections were deparaffinized with xylene then rehydrated in descending alcohol series. The sections were washed briefly in distilled water and stained in Harris' hematoxylin solution (Sigma-Aldrich, USA) for 10 minutes followed by washing in running water for 5 minutes. The sections were dipped in 1% acid alcohol for 30 seconds and washed in running water to remove excess dye from the sections then were dipped in ammonia water and rinsed in running water. The sections were counterstained in eosin-phloxine solution for 30-60 seconds and dehydrated through graded alcohol in ascending order and cleared in xylene. The cell nuclei should be blue but the cytoplasm is pink to red in the sections.

The severity of pulmonary fibrosis was assessed according to the method described previously [36]; a score system ranging from 0 (normal lung) to 4 (total fibrous obliteration of the field) was employed in lung samples. The mean fibrosis score was measured from that of six random individual fields in each sections.

### 2.3. Real-Time Quantitative PCR

The lung tissues were removed to extract total RNA by TRIzol reagent (Thermo, USA). cDNA was synthesized with HiFi-Script cDNA synthesis kit (CoWin Biosciences, China) according to the instructions. Real-time PCR were conducted with the SYBR Green PCR Master Mix (Thermo, USA). The concentration of primer and probe for each target gene (miR-135a and inflammatory mediators) was optimized according to the instructions. *β*-Actin gene served as a reference control. Relative gene expression were analyzed based on the 2^-*ΔΔ*t^ method [37]. For each lung tissue, the target genes were examined in triplicate. All primer sequences for target genes are shown (Table 1).

### 2.4. Immunofluorescence

Staining on lung tissue sections was conducted as previously described [38]. 10 *μ*m cryosections were blocked with 5% normal goat serum in PBS containing 0.05% Triton X-100 for 1 hour (h) at room temperature (RT). Then sections were incubated with antibodies against E-cadherin (ab11512, Abcam), vimentin (#3932, CST), or *α*-SMA (14-9760-82, Invitrogen) at 4°C overnight. After washing with PBS, the sections were incubated with FITC-conjugated secondary antibody for 1 h at room temperature. Normal serum was used as negative control. Fluorescence signals were examined using the Nikon Eclipse E600 fluorescence microscope (Nikon, Tokyo, Japan). Representative images were captured from at least 15 random regions in a blinded manner for each experiment, and the mean intensity was analyzed using the ImageJ software (available at https://rsb.info.nih.gov/ij/, Bethesda, MD, USA). The nuclei were stained with DAPI in PBS (Molecular Probes) for 10 min.

### 2.5. Western Blot

Changes in the expression levels of proteins in the mice lung tissues were examined by western blotting. Briefly, the lung tissue were homogenised in a lysis buffer (RIPA lysis buffer: 25 mM Tris-HCl pH 7.5, 150 mM NaCl, 1% NP-40, 1 mM EDTA pH 8.0 with 1 mM PMSF, 1 mM Na_3_VO_4_, and Protease Inhibitor (Cocktail-P2714, Sigma)). Protein lysate (30 ng) was subjected to SDS-PAGE gel. Then proteins were transferred from the gels to PVDF membrane. The membranes were blocked with 5% nonfat milk in TBST (20 mM Tris-HCl, 150 mM NaCl, and 0.05% tween-20) and incubated with anti-p65 antibody (ab16502, Abcam), anti-p-p65 antibody (ab86299, Abcam), anti-TLR4 antibody (19811-1-AP, ProteinTech), anti-p-IKB*α* (ab133462, Abcam), anti-IKB*α* (ab32518, Abcam), anti-p-IKK*α* (ab38515, Abcam), anti-IKK*α* (ab32041, Abcam), or anti-*β*-actin antibody (60008-1-Ig, ProteinTech) at 4°C overnight. Then, the corresponding secondary antibodies (1 : 5000, ProteinTech) conjugated with HRP were applied for 90 min at room temperature. The membranes were exposed to ECL (#6883, CST Signal Fire™ ECL). The bands were analyzed by the ImageJ software. The levels of target proteins were normalized by *β*-actin.

### 2.6. Lentivirus Vector System

pri-miR-135a or negative control sequences were inserted into the pGCsil-GFP vector (GENECHEM) to generate a lentivirus-mediated miR-135a construct (LV-miR-135a) and lentivirus control vector (LV-control). The virus packaging process was as follows. In details, the LV-miR-135a or LV-control construct (20 *μ*g) with the pHelper 1.0 packaging vector (15 *μ*g) and pHelper 2.0 envelop vector (10 *μ*g) was cotransfected into the HEK293T cells using Lipofectamine 2000 (Invitrogen). The culture medium was collected and concentrated by ultracentrifugation after 48 h of transfection. The virus were aliquoted and stored at -80°C. The final titer of lentivirus was 5 × 10^11^ vg/ml.

### 2.7. Transduction of Lentivirus in Lung Tissues

The mice (6-week old) were anesthetized by sodium pentobarbital and intratracheally instilled with 60 *μ*l LV-control or LV-miR-135a (5 × 10^11^ vg/ml). Fourteen days later, the mice were treated with silica or saline. Lung tissues were removed to be analyzed after 3 weeks following treatment.

### 2.8. BEAS-2B Cell Culture and Transfection

BEAS-2B cells were cultured in 10% FBS 1640 medium. Hsa-miR-135a mimic (50 nM) or inhibitor (100 nM) was transfected into cells with Lipofectamine 2000 (Invitrogen) according to the manufacturers' instructions for DNA plasmids.

### 2.9. Statistics

All data were expressed as the mean ± SEM. Analyses were performed using GraphPad Prism 5.0 (GraphPad Software, San Diego, CA). Differences between groups were calculated using the one-way ANOVA test. Correlation of the miR-135a expression with fibrosis severity was examined using the Pearson correlation. A *p* value < 0.05 was considered as statistical significance.

## 3. Results

### 3.1. Silica Induces Pulmonary Fibrosis in Mice

**Figure 1:**
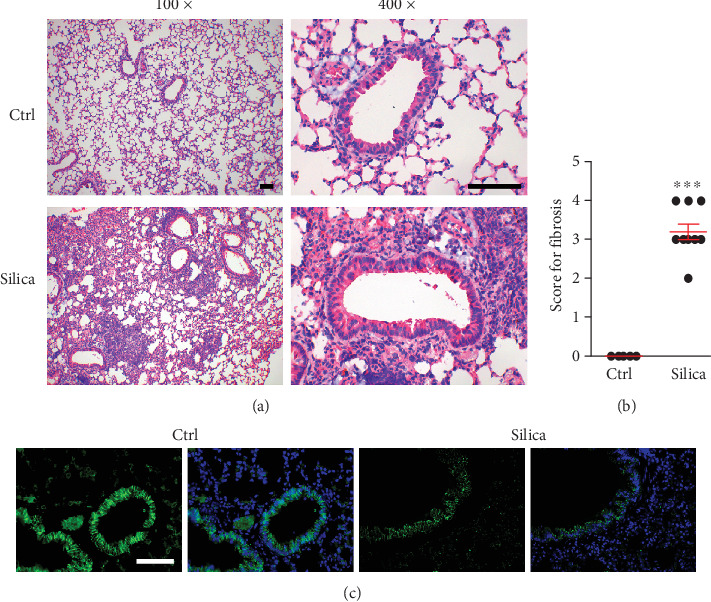
Silica induces pulmonary fibrosis in mice. (a) HE stain for lung sections 28 days following saline or silica instillation. Bar = 10 *μ*m. (b) The grading numerical scale for the severity of lung fibrosis. Scale 0 indicated the absence of fibrosis but scale 1 to 4 represented light to intense fibrosis. (c) Immunostaining of E-cadherin in lung sections of control or silica-instilled mice. Bar = 10 *μ*m. ^∗∗∗^*p* < 0.001, compared to Ctrl.

miR-135a has antifibrosis effects in the heart [15]. In order to investigate the role(s) of miR-135a in the silica-induced pulmonary fibrosis, the mice was instilled intratracheally with silica to induce pulmonary fibrosis. HE stain for lung sections exhibited much more sever fibrosis 28 days following silica instillation, when compared with control (Figure 1(a)). We employed the grading numerical scale 0-4 to determine the severity of lung fibrosis [36]. Scale 0 indicates the absence of fibrosis but scale 1 to 4 represents light to intense fibrosis. The score of fibrosis was much higher in the lung sections from mice exposed to silica than that from control mice which exhibited scale 0 for fibrosis (Figure 1(b)).

Silica caused the decrease in epithelial cells but increase in mesenchymal cells in the lungs. We found the global lower expression of E-cadherin, an epithelial cell marker (Figure 1(c)). These data indicate silica instillation results in pulmonary fibrosis.

### 3.2. Decreased Expression of miR-135a in Lung Tissues from Silica-Instilled Mice

**Figure 2:**
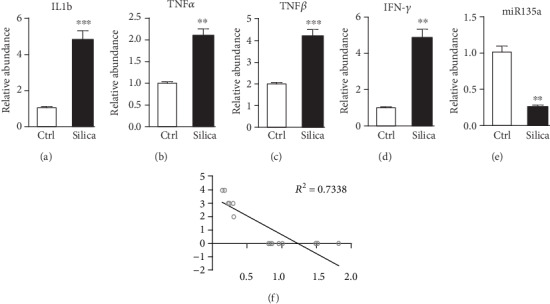
Decreased expression of miR-135a in lung tissues from silica-instilled mice. (a–d) Expression of inflammatory mediators including IL-1b (a), TNF-*α* (b), TGF-*β* (c), and IFN-*γ* (d) in lung tissues from control and silica-instilled mice using real-time QF-PCR. (e) Expression of miR-135a in lung tissues from control and silica-instilled mice using real-time QF-PCR. (f) Correlation of miR-135a expression with fibrosis score. ^∗∗^*p* < 0.01, ^∗∗∗^*p* < 0.001.

RNAs were extracted to detect the expression of miR-135a and inflammatory mediators including IL-1b, TNF-*α*, TGF-*β*, and IFN-*γ* in lung tissues from control and silica-instilled mice using real-time quantitative fluorescence PCR (QF-PCR). It was observed that the levels of IL-1, TNF-*α*, TGF-*β*, and IFN-*γ* were significantly upregulated in silica-instilled lungs (Figures 2(a)–2(d)), indicating silica-induced inflammatory responses in lung fibrosis. However, expression of miR-135a was much lower in lung tissue following silica exposure, when compared to controls (Figure 2(e)). Moreover, miR-135a expression showed high negative correlation with score levels for fibrosis in lung tissues following silica instillation (Figure 2(f)). These data indicate miR-135a probably has inhibition effects on the silica-induced lung fibrosis.

### 3.3. miR-135a Restoration in Lung Tissues Attenuates Pulmonary Fibrosis

**Figure 3:**
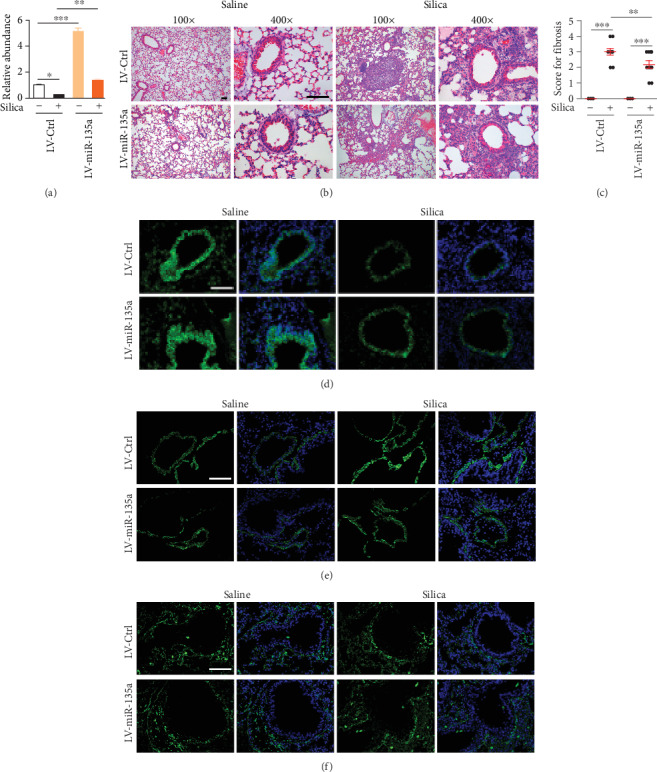
miR-135a restoration in lung tissues attenuates pulmonary fibrosis. (a) The expression of miR-135a in lung tissues from mice instilled with LV-control or LV-miR-135a following saline or silica exposure using real-time QF-PCR. (b) HE stain for lung sections from mice instilled with LV-control or LV-miR-135a following saline or silica exposure. Bar = 10 *μ*m. (c) Fibrosis score of mice instilled with LV-control or LV-miR-135a following saline or silica exposure. (d–f) Immunostaining of E-cadherin (d), *α*-SMA (e), or vimentin (f) in lung sections from mice after instillation of LV-control or LV-miR-135a following saline or silica exposure. Bar = 10 *μ*m. ^∗^*p* < 0.05, ^∗∗^*p* < 0.01, ^∗∗∗^*p* < 0.001.

Since silica instillation significantly decreased miR-135a expression in lungs and it probably suppressed the silica-induced pulmonary fibrosis, we next overexpressed miR-135a in the lungs through intratracheal instillation of lentivirus carrying either miR-135a or control gene to determine the roles of miR-135a in pulmonary fibrosis. The mice were exposed to silica 14 days after lentivirus instillation. Lung tissues were analyzed 21 days following silica exposure. miR-135a level in lungs was dramatically increased after instillation of LV-miR-135a, compared to LV-Ctrl instillation and miR-135a expression was downregulated following silica exposure (Figure 3(a)). HE stain revealed severe silica-induced fibrosis in lung sections from mice overexpressed control gene. But silica-induced fibrosis was alleviated in the lungs of mice overexpressed miR-135a (Figure 3(b)), which were consistent with the quantitative data showing a lower score of fibrosis in miR-135a-overexpressed mice, when compared to controls mice following silica exposure (Figure 3(c)).

Fibrosis was further evaluated by the expression of markers for epithelial cells or mesenchymal cells in lung sections. Restoration of miR-135a elevated E-cadherin expression and lessened *α*-SMA expression following silica instillation (Figures 3(d) and 3(e)). However, vimentin showed no obvious change after silica exposure (Figure 3(f)). These collective data indicate miR-135a restoration alleviates silica-induced pulmonary fibrosis.

### 3.4. miR-135a Restoration in Lung Tissues Attenuates Inflammatory Response in Silica-Induced Pulmonary Fibrosis

**Figure 4:**
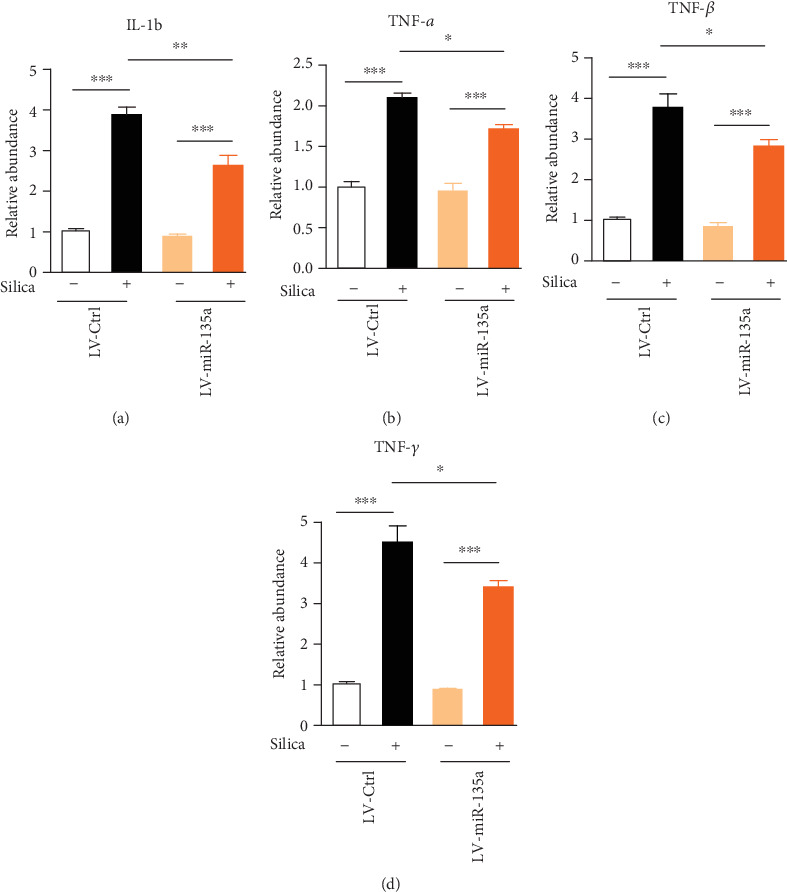
miR-135a restoration in lung tissues attenuates inflammatory response in silica-induced pulmonary fibrosis. (a–d) The expression of inflammatory mediators including IL-1b (a), TNF-*α* (b), TGF-*β* (c), and IFN-*γ* (d) in lung tissues from mice instilled with LV-control or LV-miR-135a following saline or silica exposure using real-time QF-PCR. ^∗^*p* < 0.05, ^∗∗^*p* < 0.01, ^∗∗∗^*p* < 0.001.

Expression of inflammatory mediators was further monitored by QF-PCR. Consistently, expressions of IL-1b, TNF-*α*, TGF-*β*, and IFN-*γ* were dramatically increased following silica, but their expression showed much weaker in miR-135a-overexpressed lungs, compared to the control mice following silica exposure (Figure 4), indicating miR-135a restoration attenuates inflammatory response in silica-induced pulmonary fibrosis.

### 3.5. miR-135a Restoration in Lung Tissues Inhibits NF-*κ*B Signal in Silica-Induced Pulmonary Fibrosis

**Figure 5:**
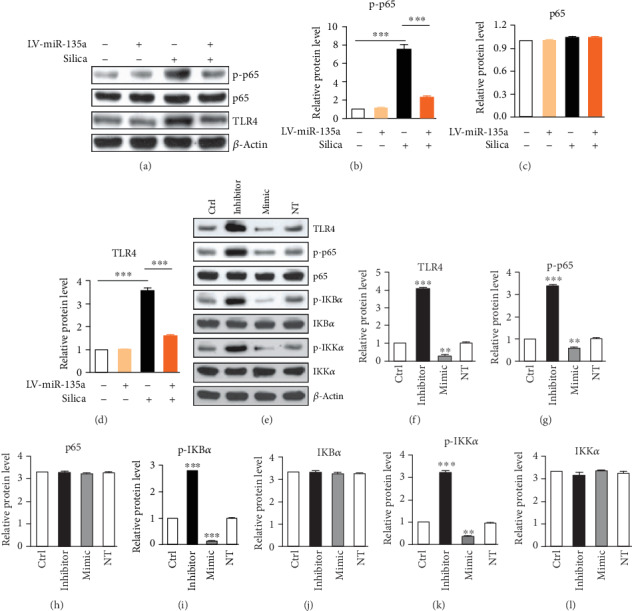
miR-135a restoration in lung tissues inhibits NF-*κ*B signal in silica-induced pulmonary fibrosis. (a) Expression of p-p65, p65, and TLR4 in lung tissues from mice instilled with LV-control or LV-miR-135a following saline or silica exposure by WB. (b–d) Quantification of p-p65 (b), p65 (c), or TLR4 (d) expression from (a). *n* = 3. ^∗∗∗^*p* < 0.001. (e) Expression of TLR4 and NF-*κ*B components in BEAS-2B cells transfected with miR-135a mimic or inhibitor. (f–l) Quantitative expression of TLR4 and NF-*κ*B components in (e). *n* = 3, ^∗∗^*p* < 0.01, ^∗∗∗^*p* < 0.001, when compared to Ctrl.

The NF-*κ*B signaling pathway aggravates inflammatory reactions in silica-induced fibrosis; WB results showed an increase in p-p65, one component of NF-*κ*B complex, in lungs after silica exposure. miR-135a overexpression reduced the p-p65 level following silica instillation, compared to control mice (Figures 5(a)–5(c)). Activation of TLR4 (Toll-like receptor 4) results in an increase of p-p65, and silica exposure led to an increase in TLR4, but miR-135a overexpression decreased TLR4 level (Figures 5(a) and 5(d)). Although there were no predicted target sites of miR-135a in 3′UTR of TLR4 or NF-*κ*B components using the TargetScan Release 6.0, we applied gain- and loss-of-function experimental strategies to investigate whether miR-135a regulates the expression of TLR4 and NF-*κ*B components. Transfection of miR-135a inhibitor into BEAS-2B cells to silence endogenous miR-135a upregulated the TLR4 protein level and enhanced the activation of the NF-*κ*B pathway. In contrast, transfection of hsa-miR-135a into BEAS-2B cells had opposite effects on TLR4 protein and NF-*κ*B activation (Figures 5(e)–5(l)). These data together indicate miR-135a restrains NF-*κ*B activation probably through targeting TLR4 in silica-induced pulmonary fibrosis.

### 3.6. Inhibition of NF-*κ*B Improves miR-135a-Mediated Protective Effects on Silica-Induced Pulmonary Fibrosis

**Figure 6:**
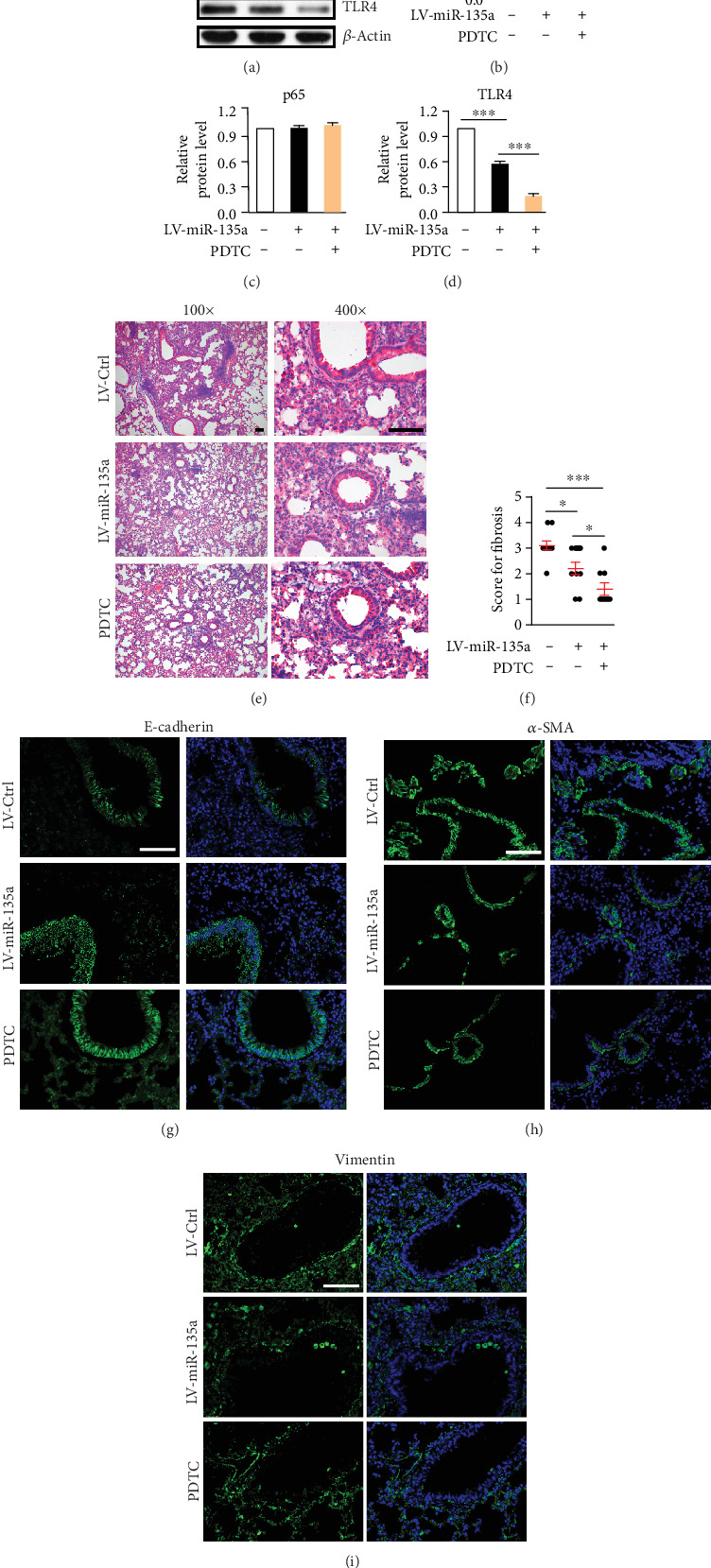
Inhibition of NF-*κ*B improves miR-135a-mediated protective effects on silica-induced pulmonary fibrosis. (a) Expression of p-p65, p65, and TLR4 in lung tissues from mice instilled with LV-control or LV-miR-135a combined with PDTC treatment following silica exposure by WB. (b–d) Quantification of p-p65 (b), p65 (c), or TLR4 (d) expression from (a). *n* = 3. (E) HE stain for lung sections from mice instilled with LV-control or LV-miR-135a combined with PDTC treatment 28 days following silica exposure. Bar = 10 *μ*m. (F) Fibrosis score of mice instilled with LV-control or LV-miR-135a combined with PDTC treatment 28 days following silica exposure. (g–i) Immunostaining of E-cadherin (g), *α*-SMA (h), or vimentin (i) in lung sections from mice instilled with LV-control or LV-miR-135a combined with PDTC treatment 28 days following silica exposure. Bar = 10 *μ*m. ^∗^*p* < 0.05, ^∗∗∗^*p* < 0.001.

Since NF-*κ*B activation facilitates the silica-induced inflammation and fibrosis, we further investigated whether miR-135a weakened inflammation and fibrosis through the regulation of NF-*κ*B activation. Silica-induced fibrosis were detected in miR-135a-overexpressed mice after intraperitoneal injection of PDTC, an inhibitor of NF-*κ*B signaling. The level of p-p65 and TLR4 showed much lower in mice when miR-135a overexpression was combined with PDTC treatment, compared to saline group (Figures 6(a)–6(d)). Consistently, miR-135a overexpression inhibited silica-induced fibrosis. PDTC treatment further hindered the fibrosis development in miR-135a-overexpressed mice (Figures 6(e) and 6(f)). And inhibition of PDTC on the fibrosis was consistent with the further elevated expression of E-cadherin but further depressed the level of *α*-SMA in miR-135a-overexpressed mice with PDTC injection (Figures 6(g) and 6(h)). PDTC injection had no effect on vimentin expression (Figure 6(i)). Therefore, inhibition of NF-*κ*B improves miR-135a-mediated protective effects on silica-induced pulmonary fibrosis.

### 3.7. Inhibition of NF-*κ*B Improves the Anti-Inflammatory Effect of miR-135a in Silica-Induced Pulmonary Fibrosis

**Figure 7:**
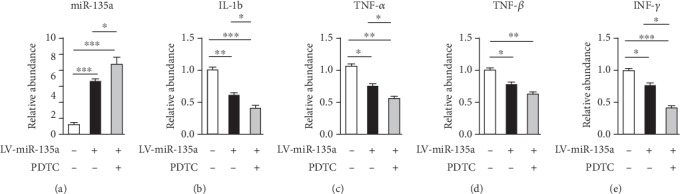
Inhibition of NF-*κ*B improves the anti-inflammatory effect of miR-135a in silica-induced pulmonary fibrosis. (a) Expression of miR-135a in lung tissues from mice instilled with LV-control or LV-miR-135a combined with PDTC treatment 28 days following silica exposure using real-time QF-PCR. (b–e) Expression of inflammatory mediators including IL-1b (b), TNF-*α* (c), TGF-*β* (d), and IFN-*γ* (e) in lung tissues from mice instilled with LV-control or LV-miR-135a combined with PDTC treatment 28 days following silica exposure using real-time QF-PCR. ^∗^*p* < 0.05, ^∗∗^*p* < 0.01, ^∗∗∗^*p* < 0.001.

Silica-induced inflammation was also detected in miR-135a-overexpressed mice after intraperitoneal injection of PDTC. PDTC treatment elevated the miR-135a expression (Figure 7(a)). The expression of inflammatory mediators, IL-1b, TNF-*α*, TGF-*β*, and IFN-*γ* were weakened in miR-135a-overexpressed mice following silica exposure. Their expression levels were further reduced after PDTC injection in miR-135a-overexpressed mice (Figures 7(b)–7(e)), indicating inhibition of NF-*κ*B enhances miR-135a-mediated amelioration of inflammation and pulmonary fibrosis induced by silica. Taken together, these collective data indicate that miR-135a probably improves pulmonary fibrosis and inflammatory responses through the regulation of the NF-*κ*B pathway. Silica-induced decrease in the expression of miR-135a disrupts its protective effects on the inflammation and pulmonary fibrosis.

## 4. Discussion

In this study, we identified miR-135a as a protective factor to alleviate silica-induced inflammation responses and pulmonary fibrosis through inhibition of NF-*κ*B activation. Silica exposure resulted in the reduction of miR-135a expression to restrain the protective pathway.

Silica exposure leads to inflammatory reactions which aggravates the abnormal synthesis and deposition of collagen in the lung. There is lack of effective treatment on the lung fibrosis after silica exposure, which prompts scientists to focus on exploring the molecular mechanisms of fibrosis development. miRNAs mediate mRNA translational repression or destabilization and regulate gene expression. miRNAs show a spatial specific expression. In recent years, miRNAs are considered as new targets for fibrosis in various tissues. Let-7i suppresses the expression of interleukin-6 (IL-6) and collagens to inhibit angiotensin II-induced cardiac fibrosis [39]. miR-125b and miR-22 are significantly downregulated in liver tissues from patients with liver fibrosis [40]. miRNAs are also reported to be involved in the pathogenesis of various pulmonary diseases [41]. miR-29 prevents the phosphorylation of PI3K-AKT induced by TGF-*β*1 and reduces extracellular matrix synthesis in human lung fibroblasts [42]. Let-7d is downregulated in lungs from patients with idiopathic pulmonary fibrosis (IPF). Inhibition of Let-7d triggers epithelial to mesenchymal transition (EMT) [43].

miR-135a has shown the protective effects under some conditions. Our study showed miR-135a expression was decreased and restoration of miR-135a improved the pulmonary fibrosis. miR-135a also suppresses the infiltration of eosinophils and mast cells into the nasal mucosa and alleviates allergen-induced inflammation [44]. Moreover, miR-135a overexpression protects human umbilical vein endothelial cell (HUVEC) injury induced by mechanical stretch [45]. miR-135a targets NHE9 to limit the growth and migration of glioblastoma cells [46]. However, miR-135a facilitates the development of some diseases as well. miR-135a accelerates renal fibrosis through the regulation of TRPC1, which increases the synthesis of extracellular matrix proteins in diabetic kidney injury [16]. miR-135a ablation represses pancreatic cancer growth in vivo [12]. miR-135a acts as an onco-miRNA to promote proliferation of a bladder cancer cell by targeting PHLPP2 and FOXO1 [47]. Therefore, the effects of miR-135a on diseases depend on its target genes.

Evidence supports miRNA-based strategies as a novel therapeutics treatment and prevention for some diseases. Administration of miravirsen which targets miR-122 decreases viral RNA levels in patients with chronic hepatitis C in clinical phase 2a trial, indicating amelioration of liver fibrosis [48]. A study applies lipid vehicle to deliver miR-34a to inhibit lung tumors burden in mice [49]. It is also reported that restoration of miRNA expression probably represents a novel treatment for the pathology of IPF [50]. The present study showed the delivery of miR-135a improved the silica-induced fibrosis and inflammation, indicating treatment strategy targeting miR-135a provides potential benefit for the patients. However, systemic introduction of miRNAs have side effects. Delivery of the miRNA therapeutic to the target tissue remains a big challenge.

Inflammatory response is a complex physiological reaction to noxious stimuli including silica. But its dysregulation often causes a variety of pathologies such as fibrosis. Several miRNAs, including miR-133a, miR-29, miR-221, miR-223, miR-155, and miR-652, are known to play roles in specific inflammatory diseases [40]. miR-326 regulates expression of TGF-*β*1 and other profibrotic genes such as Smad3, Ets1, and matrix metalloproteinase 9 (MMP9) in IPF [51]. Silica-induced collagen deposition causes silicosis that is directly controlled by cytokines. Silica activates NF-*κ*B that enhances the expression of TNF-*α*, one of the most important cytokines. Some miRNAs are reported to regulate NF-*κ*B. miR-892b activates NF-*κ*B activity in breast cancer cells [52]. miR-181b inhibits the activation of the NF-*κ*B signaling pathway to regulate vascular inflammation [53]. And miR-26a inhibits NF-*κ*B activity to attenuate collagen I expression in cardiac fibroblast [54]. The present study showed miR-135a restoration attenuated the level of p-p65, one component of NF-*κ*B complex in silica. But it remains unexplored how miR-135a restrains the level of p-p65 and whether miR-135a binds to mRNA of p65 or other molecules that phosphorylate p65. Although TLR4 expression was regulated by miR-135a in BEAS-2B cells, there were no predicted target sites in TLR4 or NF-*κ*B components. Therefore, it remains unexplored how miR-135a confines TLR4 level and NF-*κ*B activation. It is also unknown whether miR-135a regulates other pathways or molecules to alleviate the silica-induced pulmonary fibrosis.

Our data showed miR-135a was downregulated following silica exposure. But it is unclear how miR-135a expression is controlled in silica-induced pulmonary fibrosis. One of the oncogene, C-myc, associates the promoter region of miR-122 to control its expression [55]. Inflammatory mediators also affect miRNAs expression. IL-6 and Stat3 bind to upstream promoter region of miR-21 and increases its expression in human hepatocytes and hepatic stellate cells (HSC) [56]. TGF-*β* can suppress miR-29 or Let-7d expression [57]. miR-181b expression is regulated by TNF-*α* in endothelial cells (ECs) [53]. Evidence also shows NF-*κ*B confines miR-26a expression in cardiac fibroblast [54]. Therefore, it is worth to further investigate whether activated inflammatory mediators or NF-*κ*B regulates miR-135a expression following silica internalization.

## Figures and Tables

**Table 1:** The sequence of specific primers for target genes.

Gene	Primer
miR-135a	GGTATAGGGATTGGAGCCGTGG
IL-1*β*	FTGAAATGCCACCTTTTGACAGT
	RTTCTCCACAGCCACAATGAGT
TNF-*α*	FAGCACAGAAAGCATGATCCG
	RCACCCCGAAGTTCAGTAGACA
IFN-*γ*	FGCCACGGCACAGTCATTGA
	RTGCTGATGGCCTGATTGTCTT
TGF-*β*	FCTCCCGTGGCTTCTAGTGC
	RGCCTTAGTTTGGACAGGATCTG
*β*-Actin	FACATCCGTAAAGACCTCTATGCC
	RTACTCCTGCTTGCTGATCCAC
U6	FCTCGCTTCGGCAGCACA
	RAACGCTTCACGAATTTGCGT

## Data Availability

The data used to support the findings of this study are available from the corresponding author upon request.
